# Natural Rubber/Styrene–Butadiene Rubber Blend Composites Potentially Applied in Damping Bearings

**DOI:** 10.3390/polym16131945

**Published:** 2024-07-08

**Authors:** Saifeng Tang, Zhanxu Li, Weichong Sun, Yangling Liu, Jian Wang, Xiong Wang, Jun Lin

**Affiliations:** 1 State Key Laboratory of Alternate Electrical Power System with Renewable Energy Sources, North China Electric Power University, Beijing 102206, China; saifengtang@foxmail.com (S.T.); zhanxuli0614@163.com (Z.L.); 13811157813@163.com (W.S.); liuyangling010223@163.com (Y.L.); wangjian31791@ncepu.edu.cn (J.W.); 2 South China Institute of Environmental Sciences, Ministry of Ecology and Environment, Guangzhou 510655, China

**Keywords:** natural rubber, styrene–butadiene rubber, compression set, compression fatigue temperature rising, thermal oxidative aging, damping

## Abstract

Natural rubber (NR) composites have been widely applied in damping products to reduce harmful vibrations, while rubber with only a single composition barely meets performance requirements. In this study, rubber blend composites including various ratios of NR and styrene butadiene rubber (SBR) were prepared via the conventional mechanical blending method. The effects of the rubber components on the compression set, compression fatigue temperature rising and the thermal oxidative aging properties of the NR/SBR blend composites were investigated. Meanwhile, the dynamic mechanical thermal analyzer and rubber processing analyzer were used to characterize the dynamic viscoelasticity of the NR/SBR blend composites. It was shown that, with the increase in the SBR ratio, the vulcanization rate of the composites increased significantly, while the compression fatigue temperature rising of the composites decreased gradually from 47 °C (0% SBR ratio) to 31 °C (50% SBR ratio). The compression set of the composites remained at ~33% when the SBR ratio was no more than 20%, and increased gradually when the SBR ratio was more than 20%.

## 1. Introduction

Rubber composites are known as a type of advanced engineering material consisting of the rubber matrix, fillers and other cooperating agents [1,2,3,4]. The unique viscoelastic properties of rubber composites make them ideal for damping and vibration damping materials [5]. Currently, rubber vibration damping bearings have been widely used in many fields such as construction, transportation and instrumentation [6,7,8]. Among the various types of rubbers, natural rubber (NR) has received great attention in the field of rubber vibration damping materials due to its excellent mechanical properties and good elasticity [9,10]. However, due to its weak resistance to thermal oxidative aging, NR needs to be modified for practical applications. The blending of two or more kinds of rubber matrixes [11], modification by fillers [12,13] or a combination of the two methods [14,15] are commonly used for the preparation of high-performance NR composites. The tensile strength, compression resistance and tear resistance of the composites can be improved by combining NR with cis-butadiene rubber (BR) [16]; the processing, mechanical and dynamic properties can be improved by combining NR with styrene-butadiene rubber (SBR) [17]; and the oil resistance and processability of the composites can be improved by combining NR with nitrile butadiene rubber (NBR) [18]. Fillers commonly used in industry to fill NR are carbon black, silica and clay, which can further improve the mechanical properties of rubber composites such as abrasion resistance, tensile strength and tear resistance. As the most commonly used filler in the rubber industry, carbon black is available in a very wide variety. The particle size and the amount of carbon black added can significantly affect the mechanical properties of the rubber composite. N330 and N774 are two commonly used rubber fillers. N330 (high abrasion furnace black; HAF) is a carbon black with good reinforcing properties that can enhance the tensile properties, tear resistance, abrasion resistance and elasticity of the rubber composite. However, large amounts of N330 will significantly increase the compression set and compression fatigue temperature rising of the rubber composite, due to the small particle size of N330 carbon black. Therefore, in practice, it is necessary to use a portion of the larger particle size carbon black to meet the reinforcing effect of the rubber composite and reduce the compression set and compression fatigue temperature rising at the same time.

For rubber vibration damping bearings applied in engineering, the main failure causes of the bearings are the cracking and permanent deformation of the rubber material. Therefore, rubber composites for vibration damping require high-performance in compression set, compression fatigue temperature rising and mechanical properties in addition to the damping properties [19,20]. There have been many studies dealing with NR vibration damping composites. Qin et al. [21] developed a new processing method to prepare quaternary layered gradient composites by laminating NR and three types of ENR (epoxidation: 25%, 40% and 50%) in a certain order. The peak value of tan delta (tan *δ*) for the composites was up to 1.815 when the number of plies reached 32, with an effective damping temperature range of 69.7 °C from −24.8 °C to 44.9 °C (tan *δ* > 0.3). Zhang et al. [22] prepared natural rubber/hindered phenolic/styrene-isoprene block copolymer (NR/AO-80/SIS) composites. NR/AO-80/SIS were made of hindered phenolic polar small molecules (AO-80) and styrene–isoprene block copolymer (SIS) as damping performance enhancement components. They investigated the effects of SIS and AO-80 on the morphology, structure, mechanical properties, aging resistance and damping properties of NR/AO-80/SIS composites. The effective damping temperature range extends to a maximum of 114.5 °C, indicating that SIS significantly improves the damping properties of NR/AO-80. Although current studies have indeed prepared some high-performance NR composites, some of them are difficult to put into mass production because of the high cost of the modification or the complexity of the process. Some other NR composites show superior properties, but there is a lack of research on the properties valued by composites for vibration damping bearings. There is also a lack of basic research on the effects of combining NR with other rubbers on the properties of composites for vibration damping bearings. In general, the factors affecting the compression set of rubber composites are the elasticity of the rubber matrix, the filler (type, particle size and morphology) and the vulcanization system [23,24]. The compression fatigue temperature rising of rubber composites is also influenced by the rubber matrix (type and number of unsaturated bonds and their configuration), filler (type, particle size and morphology) and the vulcanization system [25,26]. 

Natural rubber, with its excellent mechanical properties and elasticity, is the most commonly used material for manufacturing rubber vibration damping bearings. With good processing properties and fatigue resistance, SBR is expected to compensate for the shortcomings of natural rubber. It is noted that there are few studies on the blending proportions of NR/SBR on the physical properties of rubber composites for vibration damping bearings. Therefore, in this study, NR/SBR composites with different blending proportions (100/0, 90/10, 80/20, 70/30, 60/40 and 50/50) were prepared by the mechanical blending method, which is low cost and convenient in the preparation process, and the tensile strength, tear strength, thermal oxidative aging performance, compression set, compression fatigue temperature rising and dynamic viscoelasticity of NR/SBR composites were investigated. This study has certain significance for the development of NR composites for highly durable vibration damping bearings.

## 2. Materials and Methods

### 2.1. Materials

Natural rubber (RSS1; number-average molecular weight (M_n_): 9.8 × 10^5^ g/mol; ML (1 + 4) 100 °C: 65 MU) was supplied by Yunnan Natural Rubber Industry Group Co., Kunming, Yunnan, China. SBR 1502 (M_n_: 1.1 × 10^5^ g/mol; styrene content: 23.5%; ML (1 + 4), 100 °C: 48 MU) was supplied by Guangzhou Rubber Industry Research Institute, Guangzhou, China. Carbon blacks N330 (reinforcing filler, particle size: 26~30 nm) and N774 (reinforcing filler, particle size: 80~170 nm) were supplied by Cabot (Tianjin, China) Limited. Zinc oxide (ZnO, vulcanizing activator), hydrophobic silica (reinforcing filler), stearic acid (SA, vulcanizing activator), rubber protective wax (Wax, physical antioxidant), N-(1,3-dimethylbutyl)-N-phenyl-p-phenylene diamine (4020, chemical antioxidant), 1,2-dihydro-2,2,4-trimethyl-6-ethoxyquinoline (AW, chemical antioxidant), bis(3-(triethoxysilyl) propyl) tetrasulfide (Si-69, coupling agent), rubber plasticizer (naphthene base mineral oil, for reducing interactions between rubber macromolecules), N-cyclohexylbenzothiazole-2-sulphenamide (CZ, vulcanizing agent), dithiocaprolactame (DTDC, vulcanizing agent) and sulfur (S) were purchased from the chemical reagent shop of Beijing. All materials were used as received.

The pure NR was first plasticated in a two-roll mill (XK-150, Guangdong Zhanjiang Guangyi Machinery, Co., Ltd., Zhanjiang, China) for better processing fluidity, and then the SBR was added to obtain the NR/SBR compound. To obtain NR/SBR composites, the ingredients were added into the compound through the two-roll mill in an orderly manner and were then cured in a standard mold using a hydraulic hot press (XLB-D 350, Huzhou Dongfang Machinery, Co., Ltd., Huzhou, China) at 150 °C under 15 MPa. It is worth mentioning that we added 5 phr hydrophobic silica at the early stage of two-roll mixing process, which helped increase the strength of the NR/SBR mixture, to facilitate the further addition of other components. In contrast, if no (or less than 5 phr) silica is added, the mixing of NR and SBR becomes very difficult as the mixture will be in a crumbly state and can de-roll during the mixing process.

In this work, NR/SBR composites with various proportions of NR and SBR were prepared and marked as NR/SBR-x (x represents the proportion of SBR in the rubber matrix). The recipe (phr) for the NR/SBR-x composites was as shown in Table 1.

### 2.2. Characterization and Measurements

In this study, the cure curves of the composites were tested by an MDR-2000 computer-type rotorless vulcanization (Shanghai Dengjie Machinery & Equipment Co., Shanghai, China). According to ISO 37:2024 [27], mechanical behavior was characterized through tensile tests using a GT-TC 2000 electrical tensile tester (Gotech Testing Machines (Dong Guan) Co., Ltd., Dongguan, China). Shore A hardness was tested using a rubber hardness apparatus (LX-A, Liuling Instrument, Shanghai, China). Tensile and hardness test results are the averages of five tests. The tensile fracture surfaces of the composites were observed by scanning electron microscopy (SEM, S-4800, Hitachi Co., Tokyo, Japan). Thermal oxidative aging property was characterized by the difference in mechanical properties before and after aging, in which an aging oven (401A, Shanghai Experimental Instrument Factory, Shanghai, China) was used, according to ISO 188:2023 [28], at 100 °C for 70 h to age the composites. The mechanical properties of the aged composites were tested in the same way as above. The strain sweep of the uncured compounds and cured composites was conducted using the RPA 2000 Rubber Process Analyzer (RPA, Alpha Technologies Co., Hudson, USA) at 60 °C and 1 Hz. Dynamic mechanical analysis (DMA) tests were conducted using a dynamic mechanical analyzer (TA Q800, TA Instruments, New Castle, DE, USA) from −60 °C to 60 °C at 1 Hz under the tensile mode at a heating rate of 3 °C/min and a 0.1% strain. According to ISO 815-1: 2019 [29], the compression set of an elastomer is the residual reduction in thickness after 30 min of testing. It indicates the ability of a rubber compound to maintain its elastic properties under long-term compressive loads. The compression set is important for assessing the deformation resistance of the composite material, and the deformation resistance of the material further affects the service life of the product. The compression set is determined as a percentage of the original thickness as follows [24]:

(1) $$C\;(\%)=(\frac{t_{1}-t_{2}}{t_{1}-t_{0}})\times \;100$$

where *C* (%) is the compression set; *t*_1_ = the original thickness; *t*_2_ = the thickness of the sample after removal from the device; and *t*_0_ = the thickness of the spacer bar used. The compression action goes up to 25% of the original thickness, tested at 85 °C for 22 h. Compression fatigue temperature rising was tested using a RH-2000 rubber compression heating instrument (Gotech Testing Machines (Dong Guan) Co., Ltd., Dongguan, China) according to ISO 4666-3: 2022 [30] at a temperature of 55 °C and a frequency of 30 Hz for 25 min. 

## 3. Result and Discussion

### 3.1. Curing Characteristics of NR/SBR-x Composites

The cure characteristics of the NR/SBR-x composites are shown in Table 2. Both NR and SBR are non-polar unsaturated rubbers with similar curing characteristics and can be cured in the same cure system. Maximum torque (*M_H_*), minimum torque (*M_L_*), curing time (*t*_90_), *t_s_*_2_ and *CRI* are important parameters [31]. *M_H_* is the maximum torque achieved, and it can be used to indicate the shear modulus for a rubber composite. *M_L_* is the lowest torque value, and it can be used as a measure of the rigidity and viscosity of an uncured rubber compound. Δ*M* is usually used to estimate the crosslink density of a rubber composite. The time required to reach 90% of the maximum torque is termed *t*_90_, from which the cure time of the NR/SBR-x compounds is determined to prepare the NR/SBR-x composites. The time for the torque to reach the minimum torque plus 0.2 dN·m is termed *t_s_*_2_, which reflects the operational safety of the rubber compound. The cure rate index (*CRI*) indicates the rate of the cure process for NR/SBR-x composites, and it is calculated as follows [32]:

(2) $$CRI=\frac{100}{t_{90}-t_{s2}}$$

From Table 1, it can be seen that the increase in SBR content in general leads to the decrease in both the *M_L_* and *M_H_* of the composites, indicating the decreased viscosity of uncured NR/SBR compounds and the reduced shear modulus of NR/SBR composites. It can also be observed that there is an overall trend which demonstrates that the increasing SBR content reduces the ΔM values of NR/SBR composites slightly, even though the trend shows some irregularity in the order of Δ*M* values as functions of SBR content. Such results indicate that the crosslink density is slightly lower for the NR/SBR composites with a larger SBR content. It is worth noting that the curing rate of the NR/SBR-x composites increases significantly with the increase in SBR content, which can be observed from the decrease in *t*_90_ and the increase in *CRI*. A higher vulcanization rate of the rubber compound means higher productivity, but the value of *t_s_*_2_ needs to be considered, as too short a *t_s_*_2_ may lead to scorching during production. Therefore, the vulcanization rate of the compound needs to be controlled within a reasonable range according to the actual situation.

### 3.2. Strain Sweep Analysis of Uncured NR/SBR-x Compounds

**Figure 1:**
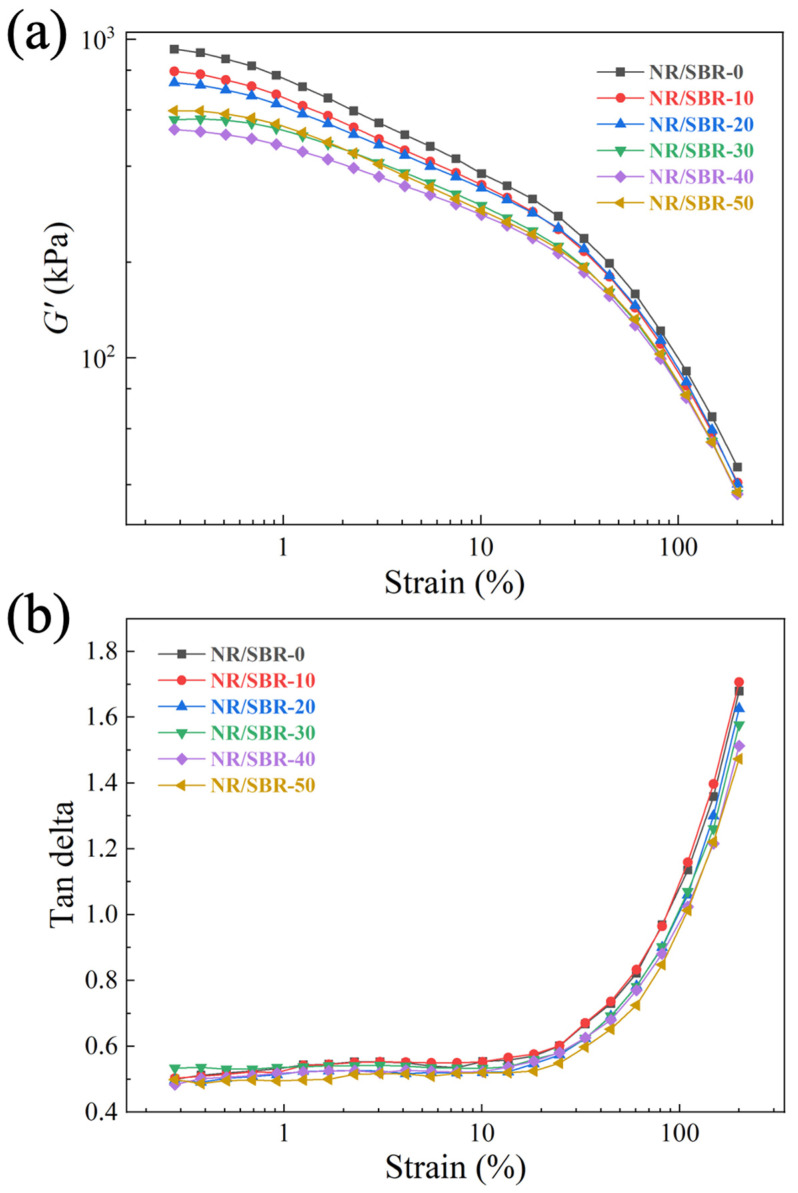
(**a**) G′-strain and (**b**) tan delta–strain curves of uncured NR/SBR-x compounds.

The storage modulus–strain curves of uncured NR/SBR-x compounds are shown in Figure 1a. It can be shown that the storage modulus (*G′*) of the NR/SBR-x compounds gradually becomes lower as the SBR content increases. This is due to the average molecular weight of NR being larger than that of SBR, which makes the *G′* of NR higher [33,34]. The average molecular weight of NR is 9.8 × 10^5^, while the average molecular weight of SBR is approximately 1.1 × 10^5^. In addition, with the gradual increase in strain, the *G′* of the compounds shows a nonlinear decreasing trend which can reflect the strength of the Payne effect and thus the structural strength of the filler network [35,36,37]. It was observed that the Payne effect of NR/SBR-x compounds decreases with the increase in SBR content.

The tan *δ*-strain curves of NR/SBR-x compounds are shown in Figure 1b. It can be observed that the tan *δ* of NR/SBR-x compounds shows an increasing trend with the increase in strain, which is due to the disruption and reorganization of the filler network. The magnitude of the tan *δ* can reflect the activity of the rubber macromolecule chains, and the tan *δ* of the compounds gradually decreases with the increase in the SBR content at the same strain. This is because the SBR macromolecule chains are not as flexible as NR due to the benzene rings contained in the SBR, which restrict the movement of the SBR chains [38].

### 3.3. Mechanical Properties and Thermal Aging Properties

**Figure 2:**
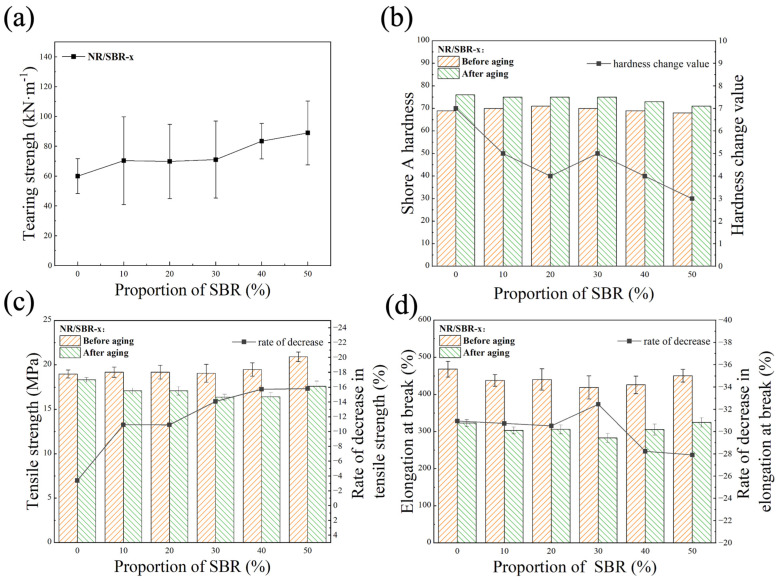
Mechanical properties of NR/SBR-x composites: (**a**) tearing strength; (**b**) shore A hardness; (**c**) tensile strength; and (**d**) elongation at break.

Figure 2a demonstrates the tearing strength of the NR/SBR-x composites, and it can be seen that the tearing strength of the composites increases with the increase in SBR content. Figure 2b shows that, before aging, the hardness of NR/SBR-x composites does not vary much with the SBR content, which may be related to the relatively high loading of carbon black (30 phr of N330 and 50 phr of N774) [39]. After aging, the hardness of all composites increases due to the increase in crosslinking degree. The change values for the hardness gradually become smaller with the increase in SBR content. This may be due to the fact that SBR contains fewer unsaturated double bonds than NR, resulting in a smaller increase in the cross-linking degree of the aged composites, which is favorable for the maintenance of the vibration damping properties of the bearings during service.

As demonstrated in Figure 2c, the tensile strength of the NR/SBR-x composites increases slightly with the increase in SBR content. As the tensile strength of pure NR (12~27 MPa) [40] is usually higher than that of pure SBR (1~2 MPa) [41], our results suggest that the filling of carbon black may strengthen the SBR phase more than the NR phase in the composites, which is likely due to the fact that carbon black tends to be distributed more in the SBR phase [42]. After aging, the rate of decrease in the tensile strength of NR/SBR-x composites increases gradually with the increase in SBR content. Since SBR is slightly more stable than NR under thermal oxidative aging, as mentioned above, such results may be tentatively attributed to the possible formation of defects between the NR and SBR phases. As will be shown below (Figure 5b), when the proportion of SBR is more than 10%, the phase separation between NR and SBR appears. However, this needs to be investigated in more detail in the future. As shown in Figure 2d, the elongation at the break of NR/SBR-x composites does not vary much with the SBR content before and after aging, which suggests that the incorporation of SBR does not affect the ductility of the composites.

### 3.4. Microstructure of Tensile Fractured Surface for NR/SBR-x Composites

**Figure 3:**
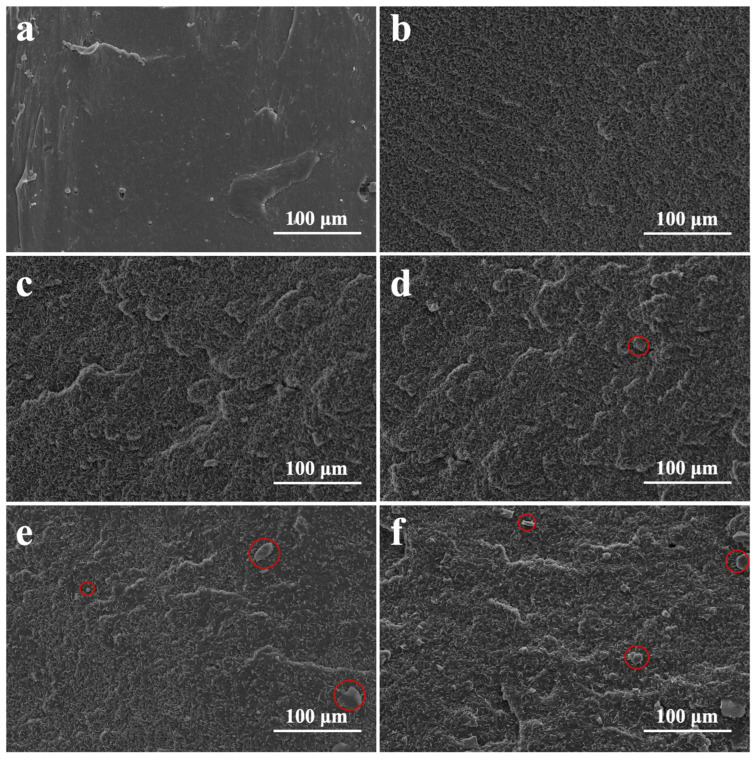
SEM images of tensile-fractured surfaces for (**a**) NR/SBR-0 composite; (**b**) NR/SBR-10 composite; (**c**) NR/SBR-20 composite; (**d**) NR/SBR-30 composite; (**e**) NR/SBR-40 composite; and (**f**) NR/SBR-50 composite.

The tensile section scans of the NR/SBR-x composites are shown in Figure 3. The composite without SBR has a relatively smooth breaking surface (Figure 3a). The breaking surface of the composites becomes rough and shows a high density of “small lamellar” bumps (Figure 3b–f), which is caused by the concurrent use of NR and SBR. Moreover, as the SBR incorporation ratio increases, the more obvious the bulges and depressions become. In addition, some carbon black aggregates (circled in red in the SEM images) appear in the composites when the SBR content is >20%, which, as mentioned throughout, is due to the fact that carbon black tends to be more distributed in the SBR phase than in the NR phase, and the higher content of carbon black in the SBR phase causes some of the carbon black to form larger aggregates.

### 3.5. Dynamic Viscoelasticity of Cured Rubber

**Figure 4:**
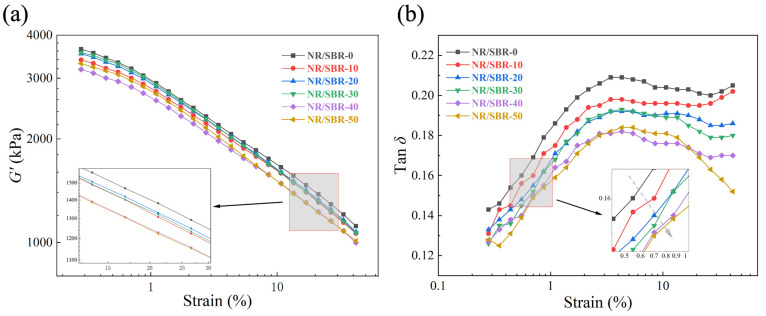
(**a**) *G′*-strain and (**b**) tan delta–strain curves of cured NR/SBR-x composites.

Figure 4a shows the *G′*-strain curves of NR/SBR-x composites, in which *G′* is affected by the filler–filler network, filler–rubber network and the crosslinked rubber network [43]. Overall, the addition of SBR results in a decrease in *G′* of the composites, which is due to the higher modulus of pure NR than that of pure SBR. Figure 4b shows that the tan *δ* of the composites gradually decreases as the proportion of SBR increases, which indicates a gradual increase in the restriction of the mobility for the rubber chains. This is due to the huge volume of phenyl groups on the SBR macromolecule which can significantly restrict the movement of molecules, and this effect becomes more obvious as the content of SBR increases.

**Figure 5:**
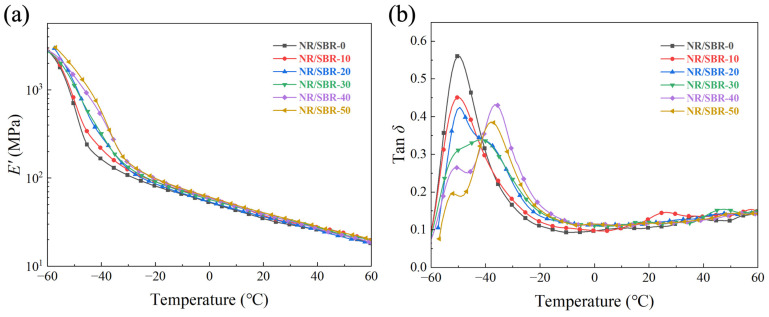
(**a**) *E′* and (**b**) tan delta versus temperature for NR/SBR-x composites.

Figure 5a shows the storage modulus (*E′*)–temperature curves of the NR/SBR-x composites. In the range of −60 °C to 0 °C, the *E′* at the same temperature increases with the increase in SBR content. This is due to the difference in glass transition temperatures (*T_g_*) between NR and SBR, with NR having a *T_g_* of about −50 °C and SBR having a *T_g_* of about −30 °C. The hardness of SBR in extreme low-temperature conditions will increase significantly when compared to the hardness at room temperature, but it still maintains good physical properties. There is little difference in the *E′* of the NR/SBR-x composites at temperatures above 0 °C.

Figure 5b shows the tan *δ*–temperature curves of the NR/SBR-x composites. The curve for NR/SBR-0 composite shows a single peak, and when the SBR content is more than 10% of the rubber matrix, the curves show two peaks. This is also due to the difference in *T_g_* of NR and SBR. With the increase in SBR content, the peak near −47 °C gradually decreases, while the peak near −32 °C gradually increases. Moreover, the decrease in the maximum value of tan delta indicates that the addition of SBR significantly enhances the restriction on the mobility of macromolecule chains.

### 3.6. Compression Properties of NR/SBR-x Composites

**Figure 6:**
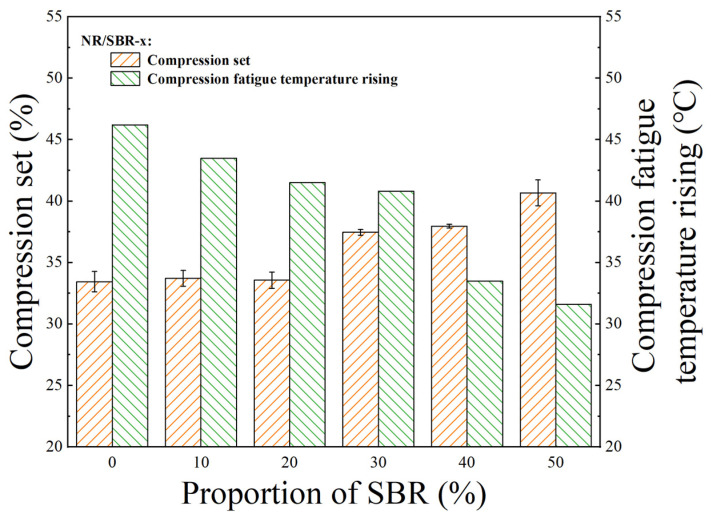
Values of compression set and compression fatigue temperature rising for NR/SBR-x composites.

In practical engineering applications, deformation failure is a common failure mode of rubber vibration damping products. Compression set is an important indicator for assessing the deformation resistance of vibration damping bearings. The heat accumulation caused by compression during the service process accelerates the thermo-oxidative aging of the composites, which seriously affects the service life of rubber vibration damping products. Therefore, the compression fatigue temperature rise also has a significant impact on the service life of the vibration damping bearings. The compression set of NR/SBR-x composites is shown in Figure 6, and it can be observed that, as the proportion of SBR increases, the compression set becomes larger in general, which is due to the phenyl group in the SBR. As the presence of phenyl restricts the mobility of the macromolecular chains, it is difficult for the macromolecular chains to return to their original positions after the pressure on the composite is withdrawn, resulting in the poor compression recovery of the composite. The observation of the data shows that when the incorporation ratio of SBR is no higher than 20%, the compression set of the composites is almost unchanged; when the incorporation ratio of SBR is higher than 20%, the compression set of the composites increases significantly. Therefore, the blending ratio of SBR and NR should be determined according to the actual required properties. 

However, it can be observed in Figure 6 that the compression fatigue temperature rising of the composites gradually decreases as the proportion of SBR is increased. The microscopic causes of dynamic heat build-up in rubber composites are mainly the friction between the rubber macromolecule chains, the friction between filler particles and rubber macromolecule chains and the friction between filler particles [44]. The motility of the SBR chains is significantly restricted due to the presence of phenyl groups, and the friction between the macromolecular chains is relatively weak. This is supported by the finding in Figure 4b that the tan *δ* of the composites decreases gradually with increasing SBR content at the same strain. At the same time, the intermolecular force of NR is smaller than that of SBR. During the test process of compression fatigue temperature rising, the friction between the rubber chains increases due to the breaking of the crosslinking network and macromolecule cleavage that tends to occur at high temperature and dynamic compression. In the application of dynamic products, if the load is large, to achieve strong resistance to compression deformation, it is necessary that a small amount of SBR (≤20%) is used; if the frequency is high, to resist fatigue temperature rising, a larger amount of SBR (>20%) can be used to extend service life.

## 4. Conclusions

In this work, the effect of NR/SBR blending proportion on the physical properties of the composites used for rubber vibration damping products was investigated. Both the cure efficiency and tearing strength of the NR/SBR blend composites increased as the content of SBR increased. The compression set of the composites increased with the higher SBR content, while the compression fatigue temperature rising decreased gradually. Such results are related to the presence of phenyl groups on the SBR macromolecule chains, which restricted the movement of the rubber chains. From the dynamic viscoelasticity analysis, tan δ gradually decreased with the increase in the SBR content, which helped to lower the compression fatigue temperature rising.

## Figures and Tables

**Table 1:** Formulations of NR/SBR-x composites. (Unit: phr).

Sample	NR/SBR-0	NR/SBR-10	NR/SBR-20	NR/SBR-30	NR/SBR-40	NR/SBR-50
NR	100	90	80	70	60	50
SBR	0	10	20	30	40	50
ZnO	5	5	5	5	5	5
hydrophobic silica	5	5	5	5	5	5
SA	2	2	2	2	2	2
Si-69	1	1	1	1	1	1
Wax	2	2	2	2	2	2
4020	2	2	2	2	2	2
AW	3	3	3	3	3	3
rubber plasticizer	5	5	5	5	5	5
N330	30	30	30	30	30	30
N774	50	50	50	50	50	50
S	1.5	1.5	1.5	1.5	1.5	1.5
DTDC	1.8	1.8	1.8	1.8	1.8	1.8
CZ	1	1	1	1	1	1

**Table 2:** Curing characteristics of NR/SBR-x composites at 150 °C.

Composites	*M_L_* (dN·m)	*M_H_* (dN·m)	Δ*M* (dN·m)	*t*_90_ (min.s)	*t*_*s*2_ (min.s)	*CRI*
NR/SBR-0	1.13	12.34	11.21	23.30	9.09	7.04
NR/SBR-10	1.05	11.96	10.91	18.52	7.54	9.11
NR/SBR-20	1.08	12.51	11.43	16.11	6.48	10.38
NR/SBR-30	0.98	12.12	11.14	16.11	7.11	11.11
NR/SBR-40	0.86	11.30	10.44	14.29	7.02	13.76
NR/SBR-50	0.86	11.03	10.17	12.04	6.33	17.51

## Data Availability

Data are contained within the article.
